# Online harms? Suicide-related online experience: a UK-wide case series study of young people who die by suicide

**DOI:** 10.1017/S0033291722001258

**Published:** 2023-07

**Authors:** C. Rodway, S. G. Tham, N. Richards, S. Ibrahim, P. Turnbull, N. Kapur, L. Appleby

**Affiliations:** National Confidential Inquiry into Suicide and Safety in Mental Health (NCISH), Centre for Mental Health and Safety, School of Health Sciences, The University of Manchester, 2nd Floor Jean McFarlane Building, Oxford Road, Manchester, M13 9PL UK

**Keywords:** Adversity, internet use, suicide, young people

## Abstract

**Background:**

Few studies have examined online experience by young people who die by suicide.

**Methods:**

A 3-year UK-wide consecutive case series of all young people aged 10–19 who died by suicide, based on national mortality data. We extracted information on the antecedents of suicide of 544 of these 595 deaths (91%) from official investigations, mainly inquests.

**Results:**

Suicide-related online experience was reported in 24% (*n* = 128/544) of suicide deaths in young people between 2014 and 2016, equivalent to 43 deaths per year, and was more common in girls than boys (OR 1.87, 95% CI 1.23–2.85, *p* = 0.003) and those identifying as LGBT (OR 2.35, 95% CI 1.10–5.05, *p* = 0.028). Searching for information about method was most common (*n* = 68, 13%), followed by posting suicidal ideas online (*n* = 57, 10%). Self-harm, bereavement (especially by suicide), social isolation, and mental and physical ill-health were more likely in those known to have suicide-related online experience compared to those who did not. 29 (5%) were bullied online, more often girls (OR 2.84, 1.34–6.04, *p* = 0.007). Online bullying often accompanied face-to-face bullying (*n* = 16/29, 67%).

**Conclusions:**

Suicide-related online experience is a common, but likely underestimated, antecedent to suicide in young people. Although its causal role is unclear, it may influence suicidality in this population. Mental health professionals should be aware that suicide-related online experience – not limited to social media – is a potential risk for young patients, and may be linked to experiences offline. For public health, wider action is required on internet regulation and support for children and their families.

## Introduction

Suicide rates in young people have risen in several high-income countries, although some countries (Australia) have experienced later rises (2009) than others (the UK, 2003) (Padmanathan, Bould, Winstone, Moran, & Gunnell, 2020). In 2019, 601 suicide deaths were registered in England and Wales in people aged 10–24, a 24% increase on the rate in 2017 (Office for National Statistics (ONS), 2020a). The rise appears to have been more marked in girls than in boys of the same age. In 2020, the suicide rate in 10–24 year olds has decreased to a level similar to that recorded in 2017 (4.8 per 100 000 population). The decrease, however, is likely to be driven by a delay in death registrations during the coronavirus disease 2019 (COVID-19) pandemic (ONS, 2021). In 2019, the suicide rate in girls and young women under 20 was the highest since recording began in 1981 (ONS, 2020a).

Previous research has highlighted several antecedents to suicide in young people (Björkenstam, Kosidou, & Björkenstam, 2017; Hawton, Saunders, & O'Connor, 2012; Hill, Witt, Rajaram, McGorry, & Robinson, 2021; Rodway et al., 2016), many of which are more common in girls than boys (e.g. family mental illness, abuse, bereavement, bullying, current or impending exams or exam results, physical health conditions, self-harm) (Rodway et al., 2020). Some of these may have contributed to the rise in suicide in young people, particularly girls. Self-harm rates in young people are certainly rising, and at a faster rate in girls than boys (McManus et al., 2019; Morgan et al., 2017). Bullying in 12–18 year olds has also risen (Ditch the Label, 2020), whilst academic stresses have recently been identified as a major source of concern for secondary and higher education students (Pascoe, Hetrick, & Parker, 2020). As suicide rates in young people have increased, there has been growing concern about the negative mental health impact of social media (HM Government, 2019) and the emotional and behavioural impact of viewing or sharing web-based self-harm imagery (Marchant, Hawton, Burns, Stewart, & John, 2021). There is also concern that exposure to internet risks (e.g. online bullying, accessing harmful/inappropriate content, enforcing negative behaviours) has increased (HM Government, 2019).

The availability and accessibility of a variety of online content has prompted concern about the potential impact on the mental health and wellbeing of children and young people. Evidence suggests possible links between harmful content exposure and poor mental health (Kelly, Zilanawala, Booker, & Sacker, 2018; Marcheselli et al., 2018; Przybylski & Bowes, 2017) and increased risk to vulnerable people who self-harm or experience suicidal thoughts or ideation (Biddle, Derges, Goldsmith, Donovan, & Gunnell, 2018; Marchant et al., 2017; Mars et al., 2015; Sueki, Yonemoto, Takeshima, & Inagaki, 2014). This concern is fuelled by media reports of suicide by young people implicating the internet, including cases of online bullying (Press Association, 2013) and exposure to self-harm images on social media (Marsh & Waterson, 2019). The effects of the internet on mental health and suicidal behaviour are, however, more complex. Digital technology can confer benefits, by offering opportunities for prevention through mutual help-seeking, crisis support, outreach by health professionals and reducing social isolation (Daine et al., 2013; Marchant et al., 2017; Mok, Jorm, & Pirkis, 2016). However risks include the potential for contagion, the exacerbation and normalisation of self-harm, and bullying (John et al., 2018; Lewis & Baker, 2011; Robertson, Skegg, Poore, Williams, & Taylor, 2012).

Young people have among the highest levels of internet use, particularly of social media. 70% of 12–15 year olds have a social media profile (Ofcom, 2019), with many using social networking sites for more than an hour per day (Booker, Skew, Kelly, & Sacker, 2015). Some studies report increased social media use tenuously predicts a decrease in the life satisfaction of 10–15 year olds (Orben, Dienlin, & Przybylski, 2019); others report increased internet use (defined as more than two, three or five hours a day or greater screen time) is associated with an increased risk of depression, self-harm and suicidal ideation in under 19 year olds (Janiri et al., 2020; Mars et al., 2020; Messias, Castro, Saini, Usman, & Peeples, 2011; Sedgwick, Epstein, Dutta, & Ougrin, 2019). Research exploring the nature of self-harm images on social media and their impact on young people also vary. In a thematic analysis of images on social media sites, there was no evidence of self-harm being sensationalised or images being used to actively encourage others to self-harm (Shanahan, Brennan, & House, 2019). However, a recent systematic review of the impact of viewing images of self-harm on young people highlights both potentially harmful (e.g. normalisation and reinforcement of self-harm) and positive (e.g. to encourage help-seeking, a source of support) impacts (Marchant et al., 2021).

Few studies have examined internet use by young people who die by suicide. Gunnell et al.'s (2012) review of coroner records found the internet contributed to a small (2%) but significant proportion of suicides in people aged over 25 (mean age 40, range 25 to 77 years). For men who died by suicide in mid-life (aged 40–54 years), suicide-related internet use was reported in 15% (NCISH, 2021a). Padmanathan et al.'s (2020) analysis of trends in suicide rates in 15–24 year olds in high-income countries found no evidence of an association between social media use (over 3 h a day) and higher youth suicide rates at a population level. However, suicide-related internet use was reported in a quarter of suicide deaths by young people in our three-year (2014–2016) UK-wide consecutive case series of people aged under 20 (Rodway et al., 2020).

Studies examining the use of the internet to search for information on suicide method have primarily focused on self-reports of self-harm. In Mars et al.'s study (2015), searching for information on suicide method was eight times more prevalent in 21 year olds with (compared to those without) a history of self-harm, with further evidence the search shaped a subsequent suicide attempt (Biddle et al., 2018). Only one study has reported, among other characteristics, the number of young Australians who died by suicide who conducted an internet search for suicide methods (3%; Hill et al., 2021). There is an apparent paucity of information on the characteristics of young people who die by suicide who used the internet for suicide-related purposes, particularly searching for information on suicide method, and the association of these searches on subsequent method choice.

In this paper, we describe detailed findings about online experiences that may have influenced suicide (including searching for information on method, visiting websites that may encourage suicide, posting suicidal ideas online and online bullying) by young people, using the personal testimony of family, friends and professionals. Our aims were to: (i) investigate suicide-related online experience among young people who die by suicide, including which experiences were the most common (ii) compare the antecedents and method of suicide in young people known to have suicide-related online experience compared to those who did not, with additional focus on the characteristics of young people known to have used the internet to obtain information on suicide method; (iii) test the hypotheses that (a) additional stresses would be reported in the lives of young people known to have suicide-related online experience, particularly if they were known to have multiple suicide-related online experiences and (b) methods of suicide would vary compared to young people who did not have suicide-related online experience.

## Methods

### Study population

In a UK-wide case series study, we examined deaths by suicide and probable suicide by young people aged 10–19 who died between 1 January 2014 and 31 December 2016. Data were collected from a range of investigations into these deaths by official bodies.

### General population mortality data

Deaths receiving a coroner (or country equivalent) conclusion of suicide (ICD-10 codes X60-X84) or undetermined intent (ICD-10 codes Y10-Y34, excluding Y33.9, Y87.0 and Y87.2) were included in the study (collectively referred to here as suicides). Narrative conclusions were also included if Office for National Statistics (ONS) procedures applied one of these ICD-10 codes. Information on these deaths were obtained from national mortality data from ONS (for deaths registered in England and Wales), National Records of Scotland (NRS; for deaths registered in Scotland) and the Northern Ireland Statistics and Research Agency (NISRA; for deaths registered in Northern Ireland).

### Data sources

#### Coroner inquest hearings/police sudden death reports

For all deaths in England and Wales, audio recordings of inquest hearings were requested from the senior coroner of the jurisdiction where the death occurred. If an audio recording was unavailable, statements or depositions submitted as evidence during the inquest were requested. In Northern Ireland, witness statements and post mortem reports were obtained from the Northern Ireland Courts and Tribunal Service (NICTS). In Scotland, redacted police death reports were requested from the Crown Office and Procurator Fiscal Service (COPFS). We obtained information from coroner inquest hearings or country equivalents for 526 (88%) deaths. For 29 deaths, data were not returned and in 40, the coroner did not wish to or was unable to provide data.

#### Child death investigations

Child death review (CDR) processes are mandatory for Safeguarding Children Partnerships (SCPs) in England for all child deaths up to the age of 18. Information is gathered from every agency that knew the child during their life and after their death via statutory CDR forms. Once completed, a multi-agency Child Death Overview Panel (CDOP) independently reviews this information and compiles it into an analysis pro forma (a ‘Form C’). We requested anonymous Form Cs from all SCPs where their respective CDOP had completed a review into a child's death by suicide or self-inflicted harm. This information is now collected in the National Child Mortality Database (NCMD). Most (119, 82%) SCPs agreed to participate. Of these, 76 provided data – resulting in Form Cs on 118 (50%) people aged under 18, 33 had not reviewed any deaths meeting our criteria in the study period, six were pending review, and data were not returned in four. 27 (18%) SCPs did not participate.

#### Case reviews

Case reviews (child safeguarding practice review in England, child practice review in Wales, case management review in Northern Ireland, and significant case review in Scotland; collectively referred to here as case reviews) are conducted when a child under the age of 18 dies or is seriously harmed and abuse or neglect is known or suspected. We searched each SCP website and the National Society for the Prevention of Cruelty to Children (NSPCC) national case review repository for any case reviews in the study period. We obtained 20.

#### Criminal justice reports

Fatal investigation reports of deaths by apparent suicide of young people in detention were obtained from the Prisons and Probation Ombudsman (PPO) website. For deaths between 1 January 2014 and 28 February 2015, the PPO notified the study of the publication of a report. For deaths after 1 March 2015, the name of the deceased remains in the report so additional notifications were not required. Deaths in prison service custody in Northern Ireland are investigated by the Prison Ombudsman for Northern Ireland and published on their website. We obtained seven criminal justice reports.

#### NCISH data

NCISH collects data on a UK-wide case series of people who die by suicide within 12 months of contact with mental health services (i.e. patients). Data are obtained in three steps, (i) national data provide information on all people who die by suicide; (ii) mental health providers identify which of these are mental health patients; (iii) clinical information is collected via a questionnaire completed by the senior professional responsible for the patient's care. Complete details of the NCISH methodology are previously published (NCISH, 2021b). NCISH data were obtained for 115 (19%) young people.

#### NHS serious incident reports

Where a suicide by a patient was identified from NCISH data, we requested a copy of the serious incident report (or critical incident review, or serious adverse incident report, referred to here as serious incident reports) from the medical director of the treating NHS Trust or Health Board. These reports describe an internal investigation of the patient's death. 97 were obtained.

### Procedures

Information on the antecedents of suicide in children and young people were recorded from the data sources on to a specifically-designed pro-forma for aggregate analysis. Information was collected about demographic and social characteristics, family environment, education, medical history, excessive alcohol use, illicit drug use, mental health history, internet use, bullying, abuse, bereavement, and service contact. These data items were applied because they have been identified in the literature as occurring before suicide in young people (Rodway et al., 2016, 2020). They were recorded if they were referred to as being present at any time in the young person's life and specifically in the three months prior to their death (referred to as ‘recent’). Reference to a specific antecedent during an official investigation implies that it was thought to be relevant to the death by the person reporting it but not necessarily causal. Further information on these variables and their definitions are shown below and in the Supplementary information (Table S1).

### Definitions

We used four variables to determine suicide-related online experience. Each of these were coded as dichotomous variables (‘Yes’ or ‘No/unknown/not reported’).

- Searching the internet for information on suicide method

This was based on information, obtained from the data sources detailed above, that described the young person as having made internet searches about suicide or suicide method prior to their death. Such information was typically reported by the police who have the legal powers to search the young person's electronic media during their investigation for the coroner.

- Visiting website(s) that may have encouraged suicidal behaviour (including chat rooms)

Information on visiting websites was also usually reported by the police during their investigation for the coroner.

- Communicating suicidal ideation or intent online

This was based on evidence of online activity that may have been reported in the police investigation or recorded from evidence from other informants (e.g. family or friends) during the coroner inquest (or in other data sources) that showed the young person had posted content about suicide or expressed suicidal ideation on social media.

- Online bullying (as a victim)

Online bullying was defined as any form of bullying, including ‘trolling’, reported to have taken place online, i.e. through social networking sites, gaming sites and messaging apps, or digital devices. This was based on witness reports from, for example, coroner inquest hearings that the young person had experienced online harassment either recently or within their lifetime.

For 41 (8%) young people, it was reported that their device(s) had been searched and no concerns on suicide-related online experience (as defined above) had been raised. In a further 14 young people (3%), devices were not searched as they were password or PIN-protected or because it was not felt to be relevant to the investigation. For 9 young people, their device or social media history was reported to have been recently deleted. These 64 cases plus a further 352 cases where suicide-related online experience was not recorded or referred to in any data source (and hence was assumed to be absent or not relevant to the young person's death) were excluded from our definition. They are referred to in this paper as young people who were not known to have any online experience that may have influenced suicide (i.e. no suicide-related online experience; *n* = 416).

### Statistical analysis

The denominator in all estimates was the total number of individuals on which at least one report was obtained (*n* = 544) unless otherwise specified. If an antecedent (i.e. bereavement) was not mentioned in any data source we assumed it was unlikely to have been present and it was recorded as being absent or not relevant to the individual death. Data were therefore coded as the presence or absence of the relevant characteristic (see online Supplementary Table S1). Logistic regression models were used to measure the estimated strength of the univariate association, adjusted for gender, age, and presence of a diagnosis, between suicide-related online experience and other demographic, social and clinical characteristics of young people who died by suicide (e.g. abuse, self-harm, alcohol or drug misuse; see online Supplementary Table S1). For the analysis of multiple suicide-related online experiences multinomial logistic regression models were fitted to compare the antecedents of people known to have had *more than one* online experience that may have influenced suicide (*n* = 35) to people known to have had a *single* suicide-related online experience (*n* = 93), and people with *no* recorded history of any suicide-related online experience (*n* = 416). Odds ratios (ORs) for the binary logistic regression and relative risk ratios (RRR) for the multinomial logistic regression with 95% confidence intervals (CIs) are presented. Stata version 14 was used for analysis. We applied ONS guidance on disclosure control to protect confidentiality, and suppressed cell counts under three, including zero. Findings for each UK nation are combined.

## Ethical standards

The study received approval from the National Research Ethics Service (NRES) Committee North West (Greater Manchester South, UK; 15/NW/0184). Permission to access confidential and identifiable information without informed consent in the interest of improving care, was obtained from the Health Research Authority Confidentiality Advisory Group (HRA-CAG; 15/CAG/0120) under Section 251 of the NHS Act 2006 and the Public Benefit and Privacy Panel for Health and Social Care (PBPP; 1617–0107).

## Results

Between 2014 and 2016, 595 people aged under 20 died by suicide in the UK, equating to a rate of 2.7 deaths by suicide per 100 000 population of 10–19 year olds. Information on the demographic, medical, psychiatric and social antecedents of suicide were obtained for 544 of these 595 young people (91%; Table 1). We obtained information from more than one data source for 184 individuals; there were no major discrepancies about antecedents between data sources. Most information was obtained from coroner inquest hearings (526, 88%). For the 18 individuals where we were unable to obtain a coroner inquest hearing, data were obtained from NHS serious incident reports (*n* = 10) and child death investigations (*n* = 8).

**Table 1.** Antecedents of suicide in young people known to have suicide-related online experience, UK (2014–2016)

Data items	Suicide-related online experience (*N* = 128)	No suicide-related online experience (*N* = 416)	Total (*N* = 544)	Analysis			
	*N* (%)	*N* (%)	*N* (%)	Unadjusted OR [95% CI]	*p* Value	Adjusted OR [95% CI]^b^	*p* Value^c^
Socio-demographic							
Gender, male	78 (61)	310 (75)	388 (71)	0.53 [0.35–0.81]	0.003	0.66 [0.42–1.02]	1.02
Ethnic minority group	14 (11)	33 (8)	47 (9)	1.43 [0.74–2.76]	0.292	1.37 [0.69–2.72]	0.369
LGBT and uncertain	14 (11)	18 (4)	32 (6)	2.72 [1.31–5.63]	0.007	2.35 [1.10–5.05]	0.028
School pupil/student	77 (60)	198 (48)	275 (51)	1.66 [1.11–2.49]	0.013	1.17 [0.73–1.86]	0.516
Employed (including apprenticeship)	30 (23)	85 (20)	115 (21)	1.19 [0.74–1.91]	0.467	2.01 [1.19–3.40]	0.009
Living alone	4 (3)	23 (6)	27 (5)	0.55 [0.19–1.62]	0.280	0.66 [0.22–1.99]	0.459
Socially isolated	27 (21)	44 (11)	71 (13)	2.26 [1.33–3.83]	0.002	2.24 [1.29–3.88]	0.004
Family history							
Mental illness	30 (23)	50 (12)	80 (15)	2.24 [1.35–3.71]	0.002	1.55 [0.91–2.65]	0.108
Physical illness	19 (15)	27 (6)	46 (8)	2.51 [1.35–4.69]	0.004	1.87 [0.97–3.58]	0.060
Alcohol and/or drug misuse	17 (13)	27 (6)	44 (8)	2.21 [1.16–4.19]	0.016	1.62 [0.83–3.15]	0.157
Witnessing domestic violence	14 (11)	22 (5)	36 (7)	2.20 [1.09–4.44]	0.028	1.52 [0.73–3.16]	0.266
Abuse and neglect							
Abuse (physical, emotional, sexual)	18 (14)	32 (8)	50 (9)	1.96 [1.06–3.63]	0.032	1.28 [0.67–2.46]	0.450
Neglect	9 (7)	14 (3)	23 (4)	2.17 [0.92–5.14]	0.078	1.69 [0.69–4.11]	0.002
Experience of bereavement							
Bereaved	45 (35)	89 (21)	134 (25)	1.99 [1.29–3.07]	0.002	1.85 [1.18–2.91]	0.008
Bereaved by suicide	22 (17)	29 (7)	51 (9)	2.77 [1.53–5.02]	0.001	2.72 [1.47–5.04]	0.001
Bullying							
Bullying (any)	50 (39)	52 (13)	102 (19)	4.49 [2.84–7.10]	<0.001	3.45 [2.13–5.59]	<0.001
Face-to-face bullying	37 (29)	52 (13)	89 (16)	2.85 [1.76–4.60]	<0.001	2.06 [1.24–3.42]	0.005
Online bullying	29 (23)	–	29 (5)	–	–	–	–
Academic pressures							
Academic pressures overall	56 (44)	118 (28)	174 (32)	1.96 [1.30–2.96]	0.001	1.51 [0.95–2.39]^d^	0.081
Current or impending exams or exam results	24 (19)	54 (13)	78 (14)	1.55 [0.91–2.62]	0.105	1.07 [0.60–1.92]^d^	0.820
Medical history							
Physical health condition	53 (41)	111 (27)	164 (30)	1.94 [1.28–2.94]	0.002	1.85 [1.21–2.84]	0.005
Excessive alcohol use	28 (22)	89 (21)	117 (22)	1.03 [0.64–1.66]	0.908	1.09 [0.66–1.80]	0.739
Illicit drug use	40 (31)	156 (38)	196 (36)	0.76 [0.50–1.16]	0.199	0.85 [0.54–1.34]	0.483
Self-harm and suicidal ideas							
Previous self-harm	87 (68)	180 (43)	267 (49)	2.78 [1.83–4.23]	<0.001	2.56 [1.62–4.09]	<0.001
Self-harm by cutting	40 (31)	79 (19)	119 (22)	1.94 [1.24–3.03]	0.004	1.51 [0.94–2.42]	0.088
Self-harm by overdose	28 (22)	50 (12)	78 (14)	2.05 [1.23–3.42]	0.006	2.00 [1.15–3.47]	0.014
Serious recent episode of self-harm (required medical treatment)	31 (24)	63 (15)	94 (17)	1.79 [1.10–2.91]	0.019	1.72 [1.01–2.92]	0.047
Suicidal intent/ideas^a^	47 (37)	220 (53)	267 (49)	0.52 [0.34–0.78]	0.002	0.39 [0.25–0.62]	<0.001
Primary diagnosis							
Any diagnosis of mental illness	62 (48)	155 (37)	217 (40)	1.58 [1.06–2.36]	0.024	1.67 [1.09–2.54]	0.017
Affective disorder (bipolar disorder and depression)	25 (20)	76 (18)	101 (19)	1.09 [0.66–1.80]	0.748	0.67 [0.36–1.24)	0.200
Anxiety/Obsessive compulsive/Post-traumatic stress disorder	13 (10)	22 (5)	35 (6)	2.02 [0.99–4.14]	0.054	1.72 [0.79–3.75]	0.171
Recent events							
Relationship break-up	34 (27)	82 (20)	116 (21)	1.47 [0.93–2.34]	0.099	1.43 [0.89–2.31]	0.142
Relationship problems	37 (29)	111 (27)	148 (27)	1.12 [0.72–1.73]	0.621	1.19 [0.76–1.89]	0.447
Housing problems	15 (12)	65 (16)	80 (15)	0.72 [0.39–1.31]	0.277	0.74 [0.40–1.38]	0.351
Workplace problems	19 (15)	64 (15)	83 (15)	0.96 [0.55–1.67]	0.882	1.28 [0.71–2.31]	0.413
Service contact (at any time)							
Mental health services (CAMHS and/or adult services)	74 (58)	167 (40)	241 (44)	2.04 [1.37–3.05]	<0.001	1.96 [1.18–3.26]	0.009
Social care or local authority services	34 (27)	65 (16)	99 (18)	1.95 [1.22–3.14]	0.006	1.49 [0.90–2.45]	0.123
Youth Offending Team or local police force	35 (27)	118 (28)	153 (28)	0.95 [0.61–1.48]	0.822	0.81 [0.51–1.30]	0.377
Looked after child	14 (11)	28 (7)	42 (8)	1.70 [0.87–3.34]	0.122	1.48 [0.73–3.01]	0.274
No service contact	36 (28)	179 (43)	215 (40)	0.52 [0.34–0.80]	0.003	0.60 [0.37–0.98]	0.043

a Excluding suicidal ideas or intent communicated online.

b Adjusted by age, gender and presence of a mental health diagnosis.

c Difference between those with and without suicide-related online experience.

d Adjusted by age, gender, presence of a mental health diagnosis and being in education (i.e. was a school pupil/student).

### Suicide-related online experience

Suicide-related online experience was reported for 128 (24%) young people, most were male (78, 61%; Table 1) and aged under 18 (77, 60%). As shown in Table 2, the commonest type of suicide-related online experience was searching for information about suicide method, followed by communicating suicidal ideas or intent online. Suicide-related online experience was more likely in girls than boys (Table 2). 29 (23%; 5% of the total sample of 544) young people were victims of online bullying – 18 (14%; 3% of the total sample) in the three months prior to death. Girls were almost three times more likely to have been bullied online than boys (Table 2). Of these 29 cases of online bullying, 16 young people had also been bullied face-to-face. There were 13 (10%; 2% of the total sample) young people who had been bullied online exclusively.

**Table 2.** Features of suicide-related online experience by gender, UK (2014–2016)

	Total sample (*N* = 544)	Boys (*N* = 388)	Girls (*N* = 156)	OR [95% CI]	*p* Value^a^
	*N* (%)	*N* (%)	*N* (%)		
Any suicide-related online experience	128 (24)	78 (20)	50 (32)	1.87 [1.23–2.85]	0.003
Searching the internet for information on suicide method	68 (13)	44 (11)	24 (15)	1.42 [0.83–2.43]	0.199
Visiting websites that may have encouraged suicide	16 (3)	11 (3)	5 (3)	1.13 [0.39–3.32]	0.817
Communicating suicidal ideas or intent online	57 (10)	36 (9)	21 (13)	1.52 [0.86–2.70]	0.152
Victim of online bullying	29 (5)	14 (4)	15 (10)	2.84 [1.34–6.04]	0.007

*Note.* The numbers do not total 128 as there was evidence of more than one type of suicide-related online experience for some young people.

a Difference between boys and girls.

### Method of suicide

**Fig. 1.**
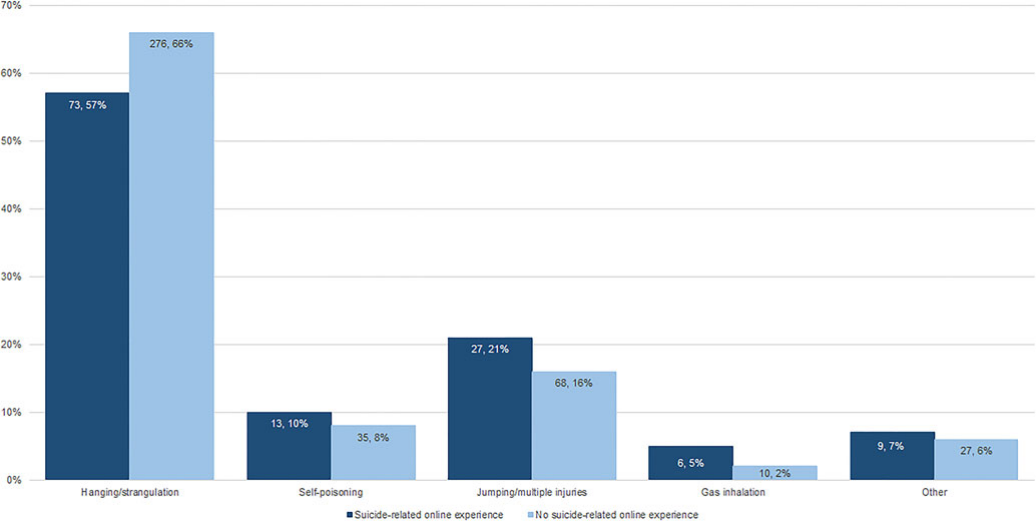
Method of suicide, UK (2014–2016).

Hanging/strangulation and multiple injuries (mainly jumping from a height or being struck by a train) were the most common methods of suicide in young people known to have suicide-related online experience (Fig. 1). However, hanging/strangulation as a method of suicide was less likely compared to young people who were not known to have suicide-related online experience (OR 0.64, 95% CI [0.42–0.98]; *p* = 0.041; Fig. 1). There were 27 (21%) deaths by multiple injuries, 13 (10%) by self-poisoning and 6 (5%) by gas inhalation – proportionally more than in young people with no suicide-related online experience (16, 8 and 2%, respectively; Fig. 1). Other methods (e.g. burning, drowning, and firearms) were infrequent (9, 7%).

### Antecedents of suicide in young people known to have suicide-related online experience

Many antecedents were more common in young people known to have suicide-related online experience than those who did not (Table 1): self-harm, social isolation, bereavement (especially by suicide), physical health problems, mental illness, and neglect. Identifying as lesbian, gay, bisexual, transgender or uncertain of their sexual identity (LGBT or uncertain), being in employment and face-to-face bullying were twice as likely among young people who were known to have suicide-related online experience.

### Obtaining information on suicide method

Of the 68 who had searched the internet for information on suicide method, 21 (31%) died by a method they had searched on – most often by hanging/strangulation (10, 15%). Eleven (16%) had searched for other methods, including the effects of non-toxic gases (*n* = 4), fatal amounts of prescribed (either for themselves or someone else) medication (*n* = 3), and how to use a firearm or jumping in front of an object or from a height (*n* = 4). Twelve (18%) died by a different method to that which was searched on – although 8 (12%) had previously self-harmed using this method. In 35 (51%), information was not recorded about the method searched on, only that a search was made.

There was no difference in the proportion of boy and girls who searched the internet for information on method (44, 11% of boys *v.* 24, 15% of girls; OR 1.17, 95% CI [0.67–2.04]; *p* = 0.585). Young people who used the internet to search for information on suicide methods had higher rates of social isolation, mental or physical illness, and self-harm (including a serious recent episode of self-harm requiring treatment), and suicidal ideas or intent. They were less likely to have had recent housing problems (see online Supplementary Table S2). Online Supplementary Table S2 also shows differences in the stresses faced by young people prior to their death by different kinds of suicide-related online experience, particularly among young people who reported being bullied online.

### Multiple suicide-related online experiences

For 35 young people (27%; 6% of all young people who died) more than one suicide-related online experience was reported (Table 3). The ‘multiple suicide-related online experience’ group were more likely than the ‘no suicide-related online experience’ group to be from an ethnic minority group, identify as LGBT or uncertain of their sexual identity, have a reported history of social isolation, face-to-face bullying, physical ill-health, self-harm (including serious recent self-harm) and mental health service contact. Rates of suicidal ideation or intent that were not communicated online or via social media, such as during a face-to-face conversation, were less likely in this group (Table 3). When compared with the ‘single suicide-related online experience’ group, they were more likely to be from an ethnic minority group, have a reported history of academic pressures and self-harm (including serious recent self-harm). They were less likely to have communicated suicidal ideation or intent. The different combinations of multiple suicide-related online experiences reported in our study are shown in online Supplementary Table S3. There were no young people who reported all four types of suicide-related online experience. However, seven young people reported three of the four types.

**Table 3.** Multinomial regression of antecedents of suicide, multiple suicide-related online experience, single suicide-related online experience and no suicide-related online experience

	Multiple SROE (*N* = 35)	Single SROE (*N* = 93)	Single SROE *v*. multiple SROE^b^		Multiple SROE *v* no SROE^c^	
	*N* (%)	*N* (%)	RRR [95% CI]	*p* Value	RRR [95% CI]	*p* Value
Socio-demographic						
Gender, male	22 (63)	56 (60)	1.15 [0.50–2.61]	0.743	0.73 [0.35–1.53]	0.399
Ethnic minority group	7 (20)	7 (8)	3.19 [1.02–9.99]	0.046	2.87 [1.13–7.25]	0.026
LGBT and uncertain	6 (17)	8 (9)	2.27 [0.72–7.18]	0.164	4.05 [1.45–11.3]	0.008
School pupil/student	16 (46)	61 (66)	0.31 [0.12–0.80]	0.016	0.50 [0.21–1.16]	0.105
Employed (including apprenticeship)	9 (26)	21 (23)	1.29 [0.48–3.45]	0.618	2.41 [0.99–5.83]	0.050
Socially isolated	11 (31)	16 (17)	2.29 [0.92–5.72]	0.075	3.95 [1.76–8.85]	0.001
Family history						
Mental illness	7 (20)	23 (25)	0.74 [0.28–1.98]	0.546	1.24 [0.50–3.10]	0.647
Physical illness	4 (11)	15 (16)	0.66 [0.20–2.17]	0.493	1.36 [0.44–4.25]	0.593
Alcohol and/or drug misuse	6 (17)	11 (12)	1.53 [0.51–4.59]	0.445	2.18 [0.81–5.84]	0.122
Witnessing domestic violence	5 (14)	9 (10)	1.59 [0.48–5.29]	0.445	2.10 [0.71–6.16]	0.178
Abuse						
Abuse (physical, sexual, emotional)	7 (20)	11 (12)	1.94 [0.66–5.71]	0.230	2.02 [0.78–5.21]	0.147
Experience of bereavement						
Bereaved	9 (26)	36 (39)	0.55 [0.23–1.32]	0.181	1.18 [0.52–2.67]	0.689
Bereaved by suicide	5 (14)	17 (18)	0.77 [0.26–2.32]	0.643	2.24 [0.79–6.36]	0.131
Bullying						
Face-to-face bullying	12 (34)	25 (27)	1.46 [0.61–3.49]	0.400	2.69 [1.21–5.95]	0.015
Academic pressures						
Academic pressures overall	18 (51)	38 (41)	2.44 [1.02–5.85]^d^	0.046	2.86 [1.30–6.26]^d^	0.009
Current or impending exams or exam results	7 (20)	17 (18)	1.80 [0.61–5.34]^d^	0.290	1.68 [0.62–4.53]^d^	0.309
Medical history						
Physical health condition	18 (51)	35 (38)	1.80 [0.81–3.96]	0.147	2.82 [1.39–5.74]	0.004
Excessive alcohol use	6 (17)	22 (24)	0.66 [0.24–1.84]	0.431	0.80 [0.31–2.04]	0.642
Illicit drug use	9 (26)	31 (33)	0.67 [0.27–1.67]	0.387	0.63 [0.28–1.44]	0.273
Self-harm and suicidal ideas						
Previous self-harm	29 (83)	58 (62)	3.47 [1.24–9.69]	0.018	6.65 [2.57–17.21]	<0.001
Serious recent episode of self-harm (required medical treatment)	13 (37)	18 (19)	3.07 [1.20–7.86]	0.020	3.73 [1.64–8.46]	0.002
Suicidal intent/ideas^a^	7 (20)	40 (43)	0.29 [0.11–0.78]	0.013	0.15 [0.06–0.38]	<0.001
Primary diagnosis						
Any diagnosis of mental illness	17 (49)	45 (48)	1.05 [0.47–2.34]	0.906	1.73 [0.84–3.54]	0.136
Affective disorder (bipolar disorder and depression)	7 (20)	18 (19)	1.05 [0.34–3.28]	0.929	0.70 [0.25–1.94]	0.488
Anxiety/obsessive compulsive/Post-traumatic stress disorder	4 (11)	9 (10)	1.23 [0.32–4.70]	0.762	1.99 [0.59–6.78]	0.267
Recent events						
Relationship break-up	9 (26)	25 (27)	0.95 [0.39–2.33]	0.917	1.38 [0.61–3.10]	0.434
Relationship problems	7 (20)	30 (32)	0.53 [0.21–1.38]	0.194	0.74 [0.31–1.78]	0.505
Workplace problems	6 (17)	13 (14)	1.34 [0.44–4.10]	0.604	1.58 [0.59–4.21]	0.359
Service contact						
Mental health services (CAMHS and/or adult services)	24 (69)	50 (54)	2.56 [0.96–6.83]	0.061	3.89 [1.61–9.42]	0.003
Social care or local authority services	9 (26)	25 (27)	0.95 [0.38–2.39]	0.919	1.43 [0.62–3.32]	0.400
Youth offending team or local police force	11 (31)	24 (26)	1.32 [0.56–3.11]	0.531	0.99 [0.46–2.12]	0.976
Looked after child	4 (11)	10 (11)	1.11 [0.32–3.90]	0.868	1.60 [0.51–5.03]	0.419
No service contact	8 (23)	28 (30)	0.63 [0.23–1.68]	0.355	0.43 [0.18–1.04]	0.061

SROE, Suicide-related online experience; RRR, Relative risk ratio.

a Excluding suicidal ideas or intent communicated online.

b Multinomial logistic regression models: single SRIU as baseline group.

c Multinomial logistic regression models: multiple SRIU as baseline group.

d Adjusted by age, gender, presence of a mental health diagnosis and being in education (i.e. was a school pupil/student).

## Discussion

In this large UK-wide study of all young people aged 10–19 who took their lives in a three-year period, to our knowledge the first of its kind, suicide-related online experience was reported in approximately 43 deaths per year. This was most often searching the internet for information on suicide method, followed by communicating suicidal ideas or intent online. 6% of young people reported multiple suicide-related online experiences, possibly indicating greater distress and potential heightened risk of suicidal behaviour. Girls were more likely to have had online experiences that may have influenced suicide than boys, despite the higher proportion of boys who died by suicide in our study. Supporting our hypotheses, several antecedents of suicide were more likely in young people known to have suicide-related online experience, compared to those who did not, including identifying as LGBT (or uncertain of their sexuality), being socially isolated, self-harm, abuse or bullying, bereavement, and having a physical or mental health condition. A number of these antecedents (e.g. identifying as LGBT, social isolation and self-harm) were also more likely to be associated with multiple compared to single suicide-related online experience. Also supporting our hypotheses, there was some evidence of a difference in suicide method between those who were known to have suicide-related online experience and those who did not; deaths by hanging were less likely among young people not known to have suicide-related online experience.

A quarter of young people who died by suicide were known to have suicide-related online experience. This is consistent with studies of clinical samples of young people presenting to hospital for self-harm who report using the internet in connection with their presentation – mainly to research suicide methods (26%, Padmanathan et al., 2018) - and with self-report surveys of young adults in the general population (23%, Mars et al., 2015), but higher than reported in older age-groups who die by suicide (2%, Gunnell et al., 2012; 15%, NCISH, 2021a). We also found 10% of young people used the internet to communicate suicidal ideas or intent; comparable to young people with a self-harm history who used the internet to discuss self-harm or suicidal feelings (9%; Mars et al., 2015). 5% of young people in our study were bullied online, particularly girls – a lower proportion than reported in earlier studies examining online bullying and self-harm in young people (13%, John et al., 2018; ~12%, Mars et al., 2020). Our findings on gender differences could be due to differences in how boys and girls use the internet. Boys spend more time online gaming and girls on social networking (Dufour et al., 2016). They are also consistent with previous longitudinal investigations that have found girls who self-harm are more likely to report suicide-related internet use overall (Mars et al., 2015), and being bullied online specifically (Mars et al., 2020) than boys.

Over a third died by a suicide method they had previously searched on. This was most often by hanging/strangulation. Few used the internet to research novel methods of suicide, despite some evidence of this in older age groups (Gunnell et al., 2012). From this study we are unable to say whether exposure to information about a specific method of suicide informed later choice – this is difficult to distinguish when the method is already common, as hanging is becoming increasingly so in young people (ONS, 2020b) – but our findings suggest for at least some young people the internet may have been a source of information about subsequent method choice. This is certainly true in what we know about young people who self-harm or attempt suicide (Biddle et al., 2012). Using the internet to search for information on suicide method may also account for why the 128 young people in our study were less likely to die by hanging/strangulation – the internet may have informed them of less violent alternatives.

### Strengths and limitations

Inquest hearings and other data sources used in this study provided rich data on factors related to suicide in young people taken from the personal testimony of families, friends and professionals. However, several limitations arise from our methodology. First, information on suicide-related online experience is likely to have been underestimated. There were 378 cases where devices had not been searched, the search history had been recently deleted, or suicide-related online experience was not referred to. Being able to determine suicide-related online experience in more cases would make the findings more robust. Furthermore, when data on a particular antecedent (e.g. abuse) was not reported in a data source (mainly coroner inquest hearings), we assumed (on the basis that coroners record factors that may have been relevant to the individual death and not factors that were absent) it was unlikely to have been present and was thus coded as ‘not present’ (i.e. not missing) for analyses. We acknowledge, however, this may under-estimate some antecedents, particularly in sensitive areas and the absence of information about an antecedent does not necessarily mean it was not present in the young person's life. As there were no missing data in our study we did not consider any sensitivity analyses. Second, we did not collect information on whether young people had accessed positive or helpful online content relating to suicide or mental health. Although it is unlikely this information would have been recorded in the data sources we examined, we acknowledge that suicide-related online experience is complex; it may pose a risk to some vulnerable people but can provide help and support to others (Mok et al., 2016). Third, this was not a risk factor study and we did not use a control group. A controlled study would have been difficult to achieve for two reasons: (i) obtaining equivalent sources of data on suitable non-suicide controls (Hawton et al., 1998); (ii) the ethical implications in contacting families. Previous psychological autopsy studies have raised doubts about equivalence in interviewing families and others along with control families - the fact of suicide itself, the impact of suicide on disclosure and the reluctance of potential controls, all distorting any comparison (Appleby, Cooper, Amos, & Faragher, 1999). Fourth, our findings cannot be linked causally to suicide but they do tell us about the stresses young people face before they take their lives – as they are taken from personal narratives reported at inquest (or other official investigations) because they were felt to be relevant to the person's death. Fifth, information may be subject to recall, information or ascertainment bias – content detail and completeness varied because the data we used were not designed for research purposes. Cases where the young person's electronic media could not be accessed may also have been misclassified as having no suicide-related online experience. Sixth, although this was a national sample, the numbers for some analyses were small presenting a potential risk for Type II errors. Finally, although the study is UK-wide, figures are driven by the larger number of suicides in England.

### Interpretation of findings

Important steps are being taken to tackle harmful suicidal content online and on social media. The forthcoming Online Safety Bill (HM Government, 2021) was published by the government in May 2021, and is currently undergoing detailed public and parliamentary scrutiny, and the Law Commission's recent report (Law Commission, 2021) recommends a new criminal offence of encouraging or assisting serious self-harm online. Our findings suggest 24% of young people who die by suicide were known to have suicide-related online experience and, for 13%, information on suicide methods was obtained via the internet. This is a likely under-estimate of the true figure of suicide-related online experience in young people – it may have a stronger role than we report – and we also do not know to what extent it was causal. However, it seems online experience may have influenced suicide in some of the young people we examined.

From this study we cannot be sure how much online safety can achieve in reducing suicide numbers but our findings suggest there is a pervasive case for strengthening online safety through a combination of measures. Evidence-informed guidance to young people and their parents about online harms and the safe use of the internet, for example through health and well-being education lessons (Royal College of Psychiatrists, 2020), guidelines to help them communicate safely about suicide online (Robinson et al., 2018), or enquiring about online behaviour as part of assessing risk. Over half of young people who reported suicide-related online experience had been in contact with mental health services, reflecting an opportunity for professionals working with young people to enquire about online experience. Legal measures and vigilance by internet companies to moderate information available online, which young people already vulnerable to suicide through other risk factors may find distressing, triggering or, for some, may normalise suicide. Regulating the availability of online information on suicide and suicide method (given that we found searching for information on suicide method accounted for approximately 23 deaths by suicide a year) could also be an effective suicide prevention method as evidence shows that restricting access to lethal means (e.g. the control of analgesics, withdrawal and safe storage of pesticides, and barriers at frequently-used locations) is associated with a decrease in suicide (Zalsman et al., 2016).

Our findings suggest that some young people at risk of suicide are using the internet to research possible methods, communicate their suicidal thoughts and, to a limited extent, seek encouragement for their actions. Action is being taken to improve online safety for children and young people but the right balance between freedom of expression and public protection needs to be struck, whilst also acknowledging that the internet can be helpful for young people's mental health and can play a role in preventing suicide. It can provide a space to share and discuss feelings without fear of being judged and can be used to engage young people to talk more about their emotions. This may be particularly pertinent during the COVID-19 pandemic and its aftermath when young people have had to spend more time online (Ofcom, 2021).

## Supplementary material

For supplementary material accompanying this paper visit https://doi.org/10.1017/S0033291722001258. click here to view supplementary material
