# Supplementary material for: Online harms? Suicide-related online experience: a UK-wide case series study of young people who die by suicide
Source: Psychol Med. 2022 May 19;53(10):4434–45. doi: 10.1017/S0033291722001258 (PMC10388316; doi:10.1017/S0033291722001258)
Supplement: Supplementary file 1 [file S0033291722001258sup.zip › S0033291722001222sup001.docx]

**SUPPLEMENT**

**Schedule for Affective Disorders and Schizophrenia for School-Age Children (K-SADS).** The K-SADS is a clinical diagnostic interview that assesses current and lifetime psychiatric disorders from the Diagnostic and Statistical Manual of Mental Disorders. The interviews were conducted by clinical psychologists and bachelor- level research assistants after receiving 40 hours of training (didactics, mock interviewing, direct supervision). The interviews were recorded, and twenty-five percent were selected at random to assess interrater reliability. The Cohen’s kappa coefficients for depressive disorders were strong (κ = 1.00).

**Variability in Positive and Negative Affect.** To measure the variability in youth affect while accounting for the assessment-to-assessment relationships of repeated EMA surveys, the mean square successive difference (MSSD; Von Neumann et al., 1941) was calculated for NA and PA. MSSD calculates the mean squared difference between successive observations, thus providing a measure of assessment-to-assessment variability in affect, with higher values indicating more variability. One PA and one NA variability datapoint were windsorized to 3 SD of the mean.

**Psychometrics of Study Variables.** Regarding the reliability of the study measures, Cronbach’s alpha for the self-reported anhedonia measure (SHAPS) was high (alpha = 0.95). We also conducted an interrater reliability analysis for a random 25% of the KSADS interviews for the depression diagnosis and found excellent reliability (κ = 1.0). We conducted split-half reliability on the PRT task and found marginally acceptable reliability (Spearman-Brown Prophecy Coefficient; SBC = 0.67). For EMA measures of PA and NA, we conducted multi-level split half reliability and found very high reliability for both PA (SBC = 0.95) and NA (SBC = 0.92).

**Supplemental Table 1: *Associations between Anhedonia, Affect, and Neural Response to Reward and Loss, controlling for Major Depression Diagnosis***

| **Measure** | **Region of Interest** | **t** | **Cluster Size** | **Direction** | **MNI Coordinates** |
| --- | --- | --- | --- | --- | --- |
| **Reward Anticipation > Loss Anticipation** | | | | | |
| SHAPS | *No significant clusters* | | | | |
| PA | *No significant clusters* | | | | |
| PRT | *No significant clusters* | | | | |
| NA | mPFC | 4.17 | 26 | Decreased | 4 36 32 |
| **Reward Anticipation > Neutral Anticipation** | | | | | |
| SHAPS | mPFC | 4.37 | 20 | Decreased | -8 34 24 |
| PA | *No significant clusters* | | | | |
| PRT | NACC | 3.54 | 3 | Increased | -12 18 -10 |
| NA | *No significant clusters* | | | | |
| **Loss Anticipation > Neutral Anticipation** | | | | | |
| SHAPS | *No significant clusters* | | | | |
| PA | *No significant clusters* | | | | |
| PRT | NACC | 3.72 | 4 | Increased | -8 10 -8 |
| NA | *No significant clusters* | | | | |
| **Win > Loss** | | | | | |
| SHAPS | *No significant clusters* | | | | |
| PA | *No significant clusters* | | | | |
| PRT | NACC | 4.17 | 4 | Increased | -6 20 -4 |
| NA | mPFC | 4.72 | 158 | Decreased | 2 60 14 |
|  | mPFC | 4.57 | 191 | Decreased | -4 44 32 |
|  | mPFC | 4.33 | 16 | Decreased | -14 34 22 |
|  | mPFC | 4.25 | 41 | Decreased | 18 42 16 |
|  | mPFC | 4.08 | 31 | Decreased | -10 54 24 |
|  | mPFC | 3.92 | 17 | Decreased | 16 56 16 |
| **Win > Neutral** | | | | | |
| SHAPS | *No significant clusters* | | | | |
| PA | *No significant clusters* | | | | |
| PRT | *No significant clusters* | | | | |
| NA | NACC | 4.01 | 10 | Increased | -6 12 -4 |
| **Loss > Neutral** | | | | | |
| SHAPS | *No significant clusters* | | | | |
| PA | *No significant clusters* | | | | |
| PRT | *No significant clusters* | | | | |
| NA | mPFC | 4.60 | 48 | Increased | -10 42 34 |
|  | NACC | 3.82 | 4 | Decreased | 6 14 -6 |

***Note.*** To test for whether primary results were robust to controlling for depression, analyses were conducted while controlling for current diagnosis of Major Depressive Disorder from the clinical interview. As with the primary analyses, additional covariates included age, sex, pubertal status, and scanner type. PA= positive affect; NA= negative affect; PRT= probabilistic reward task; mPFC = medial prefrontal cortex region of interest; NACC= nucleus accumbens region of interest.

**Supplemental Table 2: *Associations between Anhedonia, Affect, and Insula Response to Reward and Loss***

| **Measure** | **Region of Interest** | **t** | **Cluster Size** | **Direction** | **MNI Coordinates** |
| --- | --- | --- | --- | --- | --- |
| **Reward Anticipation > Loss Anticipation** | | | | | |
| SHAPS | *No significant clusters* | | | | |
| PA | *No significant clusters* | | | | |
| PRT | *No significant clusters* | | | | |
| NA | *No significant clusters* | | | | |
| **Reward Anticipation > Neutral Anticipation** | | | | | |
| SHAPS | *No significant clusters* | | | | |
| PA | *No significant clusters* | | | | |
| PRT | *No significant clusters* | | | | |
| NA | *No significant clusters* | | | | |
| **Loss Anticipation > Neutral Anticipation** | | | | | |
| SHAPS | *No significant clusters* | | | | |
| PA | *No significant clusters* | | | | |
| PRT | *No significant clusters* | | | | |
| NA | *No significant clusters* | | | | |
| **Win > Loss** | | | | | |
| SHAPS | *No significant clusters* | | | | |
| PA | *No significant clusters* | | | | |
| PRT | *No significant clusters* | | | | |
| NA | Insula | 4.10 | 19 | Decreased | 38 -10 16 |
|  | Insula | 3.98 | 12 | Decreased | 44 6 4 |
| **Win > Neutral** | | | | | |
| SHAPS | *No significant clusters* | | | | |
| PA | *No significant clusters* | | | | |
| PRT | *No significant clusters* | | | | |
| NA | Insula | 4.09 | 21 | Decreased | 38 0 14 |
| **Loss > Neutral** | | | | | |
| SHAPS | *No significant clusters* | | | | |
| PA | *No significant clusters* | | | | |
| PRT | *No significant clusters* | | | | |
| NA | *No significant clusters* | | | | |

***Note.*** Exploratory analyses tested for associations between primary and secondary study variables in the bilateral insula region of interest (i.e., AAL anatomical insula mask). As with the primary analyses, additional covariates included age, sex, pubertal status, and scanner type. PA= positive affect; NA= negative affect; PRT= probabilistic reward task.

***Supplemental Table 3: Exploratory Analyses of Group Differences and Variability in EMA Affect with Nucleus Accumbens and Medial Prefrontal Cortex Activity to Reward and Loss.***

| **Measure** | **Region of Interest** | **t** | **Cluster Size** | **Direction** | **MNI Coordinates** |
| --- | --- | --- | --- | --- | --- |
| **Reward Anticipation > Loss Anticipation** | | | | | |
| TD vs. ANH | *No significant clusters* | | | | |
| PA Variability | *No significant clusters* | | | | |
| NA Variability | *No significant clusters* | | | | |
| **Reward Anticipation > Neutral Anticipation** | | | | | |
| TD vs. ANH | *No significant clusters* | | | | |
| PA Variability | *No significant clusters* | | | | |
| NA Variability | *No significant clusters* | | | | |
| **Loss Anticipation > Neutral Anticipation** | | | | | |
| TD va. ANH | *No significant clusters* | | | | |
| PA Variability | *No significant clusters* | | | | |
| NA Variability | mPFC | 4.55 | 15 | Increased | -6 32 6 |
| **Win > Loss** | | | | | |
| TD vs. ANH | *No significant clusters* | | | | |
| PA Variability | *No significant clusters* | | | | |
| NA Variability | mPFC | 5.45 | 55 | Decreased | -6 54 40 |
|  | mPFC | 4.54 | 39 | Decreased | -10 56 22 |
|  | mPFC | 3.94 | 30 | Decreased | 4 36 18 |
|  | mPFC | 3.56 | 23 | Decreased | -24 44 14 |
| **Win > Neutral** | | | | | |
| TD vs. ANH | NACC | 4.10 | 4 | Increased | 10 16 -6 |
| PA Variability | *No significant clusters* | | | | |
| NA Variability | *No significant clusters* | | | | |
| **Loss > Neutral** | | | | | |
| TD vs. ANH | *No significant clusters* | | | | |
| PA Variability | *No significant clusters* | | | | |
| NA Variability | *No significant clusters* | | | | |

***Note.*** Analyses of group differences (i.e., typically developing versus anhedonic youth) and variability in EMA affect associations with neural reactivity to reward and loss in the nucleus accumbens and medial prefrontal cortex regions of interest. As with the primary analyses, additional covariates included age, sex, pubertal status, and scanner type. PA= positive affect; NA= negative affect; PRT= probabilistic reward task; mPFC = medial prefrontal cortex region of interest; NACC= nucleus accumbens region of interest.
