# Supplementary material for: Online harms? Suicide-related online experience: a UK-wide case series study of young people who die by suicide
Source: Psychol Med. 2022 May 19;53(10):4434–45. doi: 10.1017/S0033291722001258 (PMC10388316; doi:10.1017/S0033291722001258)
Supplement: Supplementary file 1 [file S0033291722001258sup.zip › S0033291722001258sup001.docx]

**Supplementary Table 1: Study variables**

| **Variable** | **Definition and ascertainment** | **Categories** |
| --- | --- | --- |
| Age | Obtained from national data providers (ONS, NRS, and NISRA). | Continuous variable |
| Sex | Obtained from national data providers (ONS, NRS, and NISRA). | - Male - Female |
| Method of suicide | Obtained from national data providers (ONS, NRS, and NISRA), based on ICD-10 code for cause of death. | - Hanging/strangulation - Self-poisoning - Drowning - Firearms - Jumping from a height/multiple injuries - Jumping/lying before a train - Jumping/lying before any other vehicle - Burning - Suffocation/asphyxiation - Gas inhalation - Other |
| Ethnic minority group | Recorded from data sources (e.g. coroner inquest, police report) | - Black African - Black Caribbean - Indian/Pakistani/Bangladeshi - Chinese - White - Mixed Race - Other - Not known/not recorded   Also coded as a categorical variable:   - Yes - No/unknown/not reported |
| LGBT status | Recorded from data sources (e.g. coroner inquests) that the person reported identifying as lesbian, gay, bisexual, transgender or uncertain of their sexuality prior to their death. | - Yes - No/unknown/not reported |
| School pupil/student | Recorded from data sources (e.g. coroner inquests) that the person was attending school or was a full-time student in higher or further education. | - Yes - No/unknown/not reported |
| Employed | Recorded from data sources (e.g. coroner inquests) that the person was in paid employed (including part-time or self-employment) or was in an employment or training scheme or an apprenticeship. | - Employed - Not employed/unknown/not reported |
| Living alone | Recorded from data sources (e.g. coroner inquests) that the person lived alone (e.g. absence of parents, other household members or roommates). | - Yes - No/unknown/not reported |
| Socially isolated | Recorded from data sources (e.g. coroner inquests) that the person had no or few friends or had demonstrated behaviour such as isolating themselves in their bedroom more than usually expected. | - Yes - No/unknown/not reported |

**Supplementary Table 2 (continued): Study variables**

| **Variable** | **Definition and ascertainment** | **Categories** |
| --- | --- | --- |
| Family history of mental illness | Recorded from data sources (e.g. coroner inquests) indicating that the person’s parent(s) (including step), carer, or sibling(s) had a history of mental illness. | - Yes - No/unknown/not reported |
| Family history of physical illness | Recorded from data sources (e.g. coroner inquests) indicating that the person’s parent(s) (including step), carer, or sibling(s) had a history of physical illness. | - Yes - No/unknown/not reported |
| Family history of alcohol and/or drug misuse | Recorded from data sources (e.g. coroner inquests) indicating that the person’s parent(s) (including step), carer, or sibling(s) had a history of alcohol and/or drug misuse. | - Yes - No/unknown/not reported |
| Witnessing domestic violence | Recorded from data sources (e.g. coroner inquests) that the person had been exposed to or witnessed domestic violence between parents (including step, or other primary caregivers). | - Yes - No/unknown/not reported |
| Abuse | Recorded from data sources (e.g. coroner inquests) that the person had been physically, emotionally, and/or sexually abused. | - Yes - No/unknown/not reported |
| Neglect | Recorded from data sources (e.g. coroner inquests) that the person had experienced neglect by a parent (including step) or caregiver. | - Yes - No/unknown/not reported |
| Bereavement | Recorded from data sources (e.g. coroner inquests) that someone known to the person had died, including a family member (son/daughter, parent, sibling, or other extended family member), friend or acquaintance, girlfriend/boyfriend, or other (including a pet). Includes death due to suicide, although this is also recorded separately. | - Yes - No/unknown/not reported |
| Suicide bereavement | Recorded from data sources (e.g. coroner inquests) that someone known to the person had died by suicide, including a family member (parent, sibling, or other extended family member), friend or acquaintance (including work colleague), or girlfriend/boyfriend. Includes description in a data source referring to “died by [method of suicide]”, “”took/ ended own life”. | - Yes - No/unknown/not reported |
| Bullying (any) | Recorded from data sources (e.g. coroner inquests) that the person had experienced verbal or physical bullying by school peers or work colleagues at any time prior to their death. | - Yes - No/unknown/not reported |

**Supplementary Table 2 (continued): Study variables**

| **Variable** | **Definition and ascertainment** | **Categories** |
| --- | --- | --- |
| Academic pressures overall | Recorded from data sources (e.g. coroner inquests) that the person was experiencing difficulties with school work, [perceived] failure to meet own, teacher or parent expectations, or other non-exam academic or student-related stresses (i.e. struggling with assignment(s), unhappy with course). | - Yes - No/unknown/not reported |
| Current or impending exams or exam results | Recorded from data sources (e.g. coroner inquests) that the person was anxious about current or impending exams or current or impending exam results. | - Yes - No/unknown/not reported |
| Physical health condition | Recorded from medical evidence heard during the coroner’s inquest or from other available data sources (e.g. child death investigation) that the person had a physical health condition at the time of death. | - Yes - No/unknown/not reported   If a physical health condition was reported, this was noted as free text and subsequently coded into one (or more) of the following variables:   - Circulatory - Dermatological - Digestive - Endocrine, nutritional, or metabolic - External cause of injury - Genitourinary - Musculoskeletal - Nervous system - Respiratory - Other/not elsewhere classified |
| Excessive alcohol use | Alcohol use was recorded from data sources (i.e. coroner inquests) if there was reported evidence of, e.g. alcohol consumption at a level of misuse, persistent heavy drinking, or binge drinking. | - Yes - No/unknown/not reported |
| Illicit drug use | Drug use was recorded from data sources (i.e. coroner inquests) if there was any reported evidence of the consumption of illicit drugs including heroin or other opiates, stimulants (e.g. amphetamines, LSD/mushrooms, crack/cocaine, ecstasy), benzodiazepines (excluding prescribed), cannabis, or novel psychoactive substances at any time prior to death. | - Yes - No/unknown/not reported |

**Supplementary Table 2 (continued): Study variables**

| **Variable** | **Definition and ascertainment** | **Categories** |
| --- | --- | --- |
| Previous self-harm | Recorded from data sources that the person had self-harmed (including suicide attempt), irrespective of intent, in their lifetime. Includes reported evidence of presenting to healthcare services (i.e. GP, A&E) for self-harm, descriptions from informants of previous self-harm or suicide attempts, and indirect evidence from autopsy reports (e.g. evidence of wounds or healed wounds to wrist or consistent with cutting). | - Yes - No/unknown/not reported |
| Serious recent episode of self-harm | Recorded from medical evidence heard during the coroner inquest, NCISH data or an NHS serious incident report that the last episode of self-harm prior to death required medical treatment from a GP or in hospital. | - Yes - No/unknown/not reported |
| Suicidal intent/ideas | Recorded from data sources that the person had expressed suicidal ideation or indicated that they wanted to take their own life. This included written (excluding in a suicide note) or verbal communication, text and email communication, and content about suicidal ideation posted on social media (also recorded separately). | - Yes - No/unknown/not reported |
| Diagnosis of mental illness | Recorded from medical evidence heard during the coroner inquest (e.g. from a GP or consultant psychiatrist), NCISH data or an NHS serious incident report that the person had a diagnosis of mental illness prior to their death. | - Schizophrenia and other delusional disorders - Bipolar affective disorder - Depression - Anxiety disorder - OCD - PTSD - Eating disorder - Alcohol dependence/ misuse - Drug dependence/misuse - Personality disorder - Adjustment disorder - Learning disability - Pervasive developmental disorder - Autistic spectrum disorder - ADHD - Mental disorder present but unable to specify - Other - No mental disorder - Unknown/not reported   Also coded as a dichotomous variable:   - Yes - No/unknown/not reported |

**Supplementary Table 2 (continued): Study variables**

| **Variable** | **Definition and ascertainment** | **Categories** |
| --- | --- | --- |
| Diagnosis of affective disorder | Recorded from medical evidence heard during the coroner inquest (e.g. from a GP or consultant psychiatrist), NCISH data or an NHS serious incident report that the person had a diagnosis of bipolar disorder or depression prior to their death. | - Yes - No/unknown/not reported |
| Diagnosis of anxiety/OCD/PTSD | Recorded from medical evidence heard during the coroner inquest (e.g. from a GP or consultant psychiatrist), NCISH data or an NHS serious incident report that the person had a diagnosis of anxiety, obsessive compulsive and/or post-traumatic stress disorder prior to their death. | - Yes - No/unknown/not reported |
| Relationship break-up | Recorded from data sources that the person had a relationship breakup with their partner (whether co-habiting or not) in the 3 months prior to their death. Includes boyfriend/girlfriend relationships (including online). | - Yes - No/unknown/not reported |
| Relationship problems | Recorded from data sources that the person experienced recent arguments with a current or ex-partner, being in an on/off relationship or difficulties with a relationship in the 3 months prior to their death. Excludes a relationship breakup. | - Yes - No/unknown/not reported |
| Housing problems | Recorded from data sources that the person had experienced housing instability in the 3 months prior to death, including problems with their accommodation and/or having recently changed accommodation, including moving house. | - Yes - No/unknown/not reported |
| Workplace problems | Recorded from data sources that the person had experienced problems in the workplace such as loss of job (redundancy, quitting job, being fired), unemployment, or other workplace problems (including with colleagues). | - Yes - No/unknown/not reported |
| Mental health services | Recorded from medical evidence heard during the coroner inquest (e.g. from a GP or consultant psychiatrist), NCISH data or an NHS serious incident report that the person or other informant report that the person had previous or current contact with CAMHS and/or adult mental health services, including alcohol and drug services. | - Yes - No/unknown/not reported |

**Supplementary Table 2 (continued): Study variables**

| **Variable** | **Definition and ascertainment** | **Categories** |
| --- | --- | --- |
| Social care or local authority services | Recorded from data sources based on informant report that the person had previous or current contact with child protection services, secure local authority care or social services. | - Yes - No/unknown/not reported |
| Youth Offending Team or local police force | Recorded from data sources based on informant report that the person had previous or current contact with a Youth Offending team, with the police with as an offender or a victim of crime, or with the probation service. Includes contact with prison and youth detention services. | - Yes - No/unknown/not reported |
| Looked after child | Recorded from data sources based on informant report that the person had previously or currently been a ‘looked-after’ child or young person (e.g. in public care or under welfare grounds in a secure children’s home). | - Yes - No/unknown/not reported |

ONS = Office for National Statistics; NRS = National Records of Scotland; NISRA = Northern Ireland Statistics and Research Agency; ICD-10 = International Statistical Classification of Diseases and Health-Related Problems, 10^th^ version; LGBT = lesbian, gay, bisexual, transgender; ADHD = Attention Deficit Hyperactivity Disorder; OCD = Obsessive Compulsive Disorder; PTSD = Post Traumatic Stress Disorder; CAMHS = Child and Adolescent Mental Health Services.
