# Supplementary material for: Online harms? Suicide-related online experience: a UK-wide case series study of young people who die by suicide
Source: Psychol Med. 2022 May 19;53(10):4434–45. doi: 10.1017/S0033291722001258 (PMC10388316; doi:10.1017/S0033291722001258)
Supplement: Supplementary file 1 [file S0033291722001258sup.zip › S0033291722001258sup002.docx]

**Supplementary Table 2: Antecedents of suicide by specific types of suicide-related online experience, UK (2014-2016)**

| **Data item** | **Searching for information on suicide method (n=68)** | | | **Posting suicidal ideas online (n=57)** | | | **Visiting websites**  **(n=16)** | | | **Online bullying**  **(n=29)** | | |
| --- | --- | --- | --- | --- | --- | --- | --- | --- | --- | --- | --- | --- |
|  | **N (%)** | **Adjusted OR (95% CI)** | ***p* value** | **N (%)** | **Adjusted OR (95% CI)** | ***p* value** | **N (%)** | **Adjusted OR (95% CI)** | ***p* value** | **N (%)** | **Adjusted OR (95% CI)** | ***p* value** |
| **Socio-demographic**  Ethnic minority group | 9 (13) | 1.74 (0.79-3.84) | .168 | 8 (14) | 1.82 (0.78-4.24) | .165 | <3 | --- | --- | 3 (10) | 1.10 (0.31-3.91) | .884 |
| LGBT and uncertain | 8 (12) | 2.22 (0.93-5.27) | .072 | 11 (19) | 5.13 (2.27-11.60) | <.001 | 4 (25) | 5.70 (1.68-19.32) | .005 | <3 | --- | --- |
| School pupil/student | 40 (59) | 1.21 (0.67-2.17) | .522 | 34 (60) | 0.89 (0.46-1.73) | .740 | 10 (63) | 1.53 (0.49-4.79) | .464 | 13 (45) | 0.37 (0.14-0.96) | .040 |
| Employed (including apprenticeship) | 16 (24) | 1.66 (0.86-3.19) | .128 | 13 (23) | 1.86 (0.90-3.83) | .094 | <3 | --- | --- | 8 (28) | 2.88 (1.10-7.50) | .031 |
| Socially isolated | 18 (26) | 2.74 (1.46-5.12) | .002 | 10 (18) | 1.63 (0.77-3.47) | .205 | 5 (31) | 3.07 (1.01-9.31) | .048 | 6 (21) | 1.62 (0.61-4.28) | .332 |
| **Family history**  Mental illness | 14 (21) | 1.20 (0.61-2.35) | .601 | 11 (19) | 1.09 (0.51-2.29) | .830 | 3 (19) | 1.09 (0.29-4.13) | .896 | 10 (34) | 2.11 (0.90-4.94) | .086 |
| Physical illness | 9 (13) | 1.42 (0.64-3.15) | .388 | 9 (16) | 1.87 (0.82-4.24) | .137 | <3 | --- | --- | 4 (14) | 1.19 (0.38-3.69) | .767 |
| Substance misuse | 7 (10) | 1.05 (0.44-2.51) | .911 | 11 (19) | 2.71 (1.25-5.91) | .012 | <3 | --- | --- | 4 (14) | 1.28 (0.41-3.98) | .676 |
| Witnessing domestic violence | <3 | --- | --- | 8 (14) | 2.07 (0.86-4.97) | .104 | <3 | --- | --- | 7 (24) | 3.50 (1.33-9.25) | .011 |
| **Abuse and neglect**  Abuse (physical, emotional, sexual) | 7 (10) | 0.65 (0.28-1.55) | .334 | 10 (18) | 1.90 (0.86-4.23) | .114 | 3 (19) | 1.96 (0.51-7.60) | .330 | 6 (21) | 1.62 (0.59-4.41) | .346 |
| Neglect | <3 | --- | --- | 6 (11) | 2.91 (1.06-8.01) | .039 | <3 | --- | --- | 4 (14) | 3.10 (0.95-10.16) | .062 |
| **Experience of bereavement**  Bereaved | 19 (28) | 1.10 (0.61-1.98) | .748 | 22 (39) | 2.21 (1.21-4.01) | .010 | 4 (25) | 0.96 (0.30-3.08) | .941 | 10 (34) | 1.36 (0.60-3.11) | .460 |
| Bereaved by suicide | 8 (12) | 1.28 (0.57-2.91) | .550 | 11 (19) | 2.93 (1.36-6.28) | .006 | <3 | --- | --- | 6 (21) | 2.49 (0.93-6.72) | .070 |
| **Bullying**  Bullying (any) | 18 (26) | 1.27 (0.69-2.37) | .443 | 20 (35) | 2.22 (1.17-4.20) | .014 | 5 (31) | 1.73 (0.55-5.39) | .348 | --- | --- | --- |
| Face-to-face bullying | 18 (26) | 1.58 (0.85-2.97) | .150 | 15 (26) | 1.56 (0.79-3.08) | .204 | 5 (31) | 2.08 (0.66-6.55) | .208 | 16 (55) | 5.19 (2.30-11.72) | <.001 |
| Online bullying | <3 | --- | --- | 9 (16) | 3.60 (1.49-8.69) | .004 | <3 | --- | --- | --- | --- | --- |
| **Academic pressures**  Academic pressures overall | 35 (51) | 1.87 (0.90-3.85)^3^ | .092 | 26 (46) | 1.52 (0.71-3.27)^3^ | .280 | 8 (50) | 1.04 (0.28-3.82)^3^ | .954 | 10 (34) | 0.42 (0.12-1.48)^3^ | .176 |
| Current or impending exams or exam results | 18 (26) | 2.01 (0.98-4.11)^3^ | .055 | 10 (18) | 0.77 (0.33-1.82)^3^ | .549 | 4 (25) | 1.22 (0.30-4.97)^3^ | .776 | <3 | --- | --- |
| **Medical history**  Physical health condition | 32 (47) | 2.23 (1.32-3.76) | .003 | 19 (33) | 1.16 (0.64-2.10) | .635 | 10 (63) | 3.99 (1.41-11.27) | .009 | 13 (45) | 1.73 (0.80-3.78) | .165 |
| Excessive alcohol use | 12 (18) | 0.74 (0.37-1.45) | .376 | 14 (25) | 1.48 (0.76-2.89) | .249 | 3 (19) | 0.82 (0.22-2.99) | .762 | 6 (21) | 0.96 (0.37-2.49) | .933 |

**Supplementary Table 2 (continued): Antecedents of suicide by specific types of suicide-related online experience, UK (2014-2016)**

| **Data items** | **Searching for information on suicide method (n=68)** | | | **Posting suicidal ideas online (n=57)** | | | **Visiting websites**  **(n=16)** | | | **Online bullying**  **(n=29)** | | |
| --- | --- | --- | --- | --- | --- | --- | --- | --- | --- | --- | --- | --- |
|  | **N (%)** | **Adjusted OR (95% CI)^1^** | ***p* value** | **N (%)** | **Adjusted OR (95% CI)^1^** | ***p* value** | **N (%)** | **Adjusted OR (95% CI)^1^** | ***p* value** | **N (%)** | **Adjusted OR (95% CI)^1^** | ***p* value** |
| **Medical history continued**  Illicit drug use | 21 (31) | 0.77 (0.43-1.38) | .381 | 19 (33) | 1.17 (0.63-2.19) | .616 | 5 (31) | 0.79 (0.25-2.42) | .674 | 7 (24) | 0.61 (0.24-1.52) | .287 |
| **Self-harm and suicidal ideas**  Previous self-harm | 48 (71) | 2.61 (1.44-4.73) | .002 | 40 (70) | 3.19 (1.67-6.09) | <.001 | 11 (69) | 2.27 (0.72-7.16) | .162 | 23 (79) | 3.28 (1.23-8.75) | .018 |
| Self-harm by cutting | 19 (28) | 1.18 (0.65-2.15) | .589 | 24 (42) | 2.78 (1.52-5.08) | .001 | 4 (25) | 1.03 (0.31-3.39) | .959 | 11 (38) | 1.56 (0.69-3.55) | .283 |
| Self-harm by overdose | 19 (28) | 2.72 (1.44-5.14) | .002 | 6 (11) | 0.73 (0.29-1.83) | .504 | 5 (31) | 2.96 (0.93-9.42) | .066 | 8 (28) | 2.04 (0.81-5.15) | .131 |
| Serious recent episode of self-harm (required medical treatment) | 19 (28) | 2.00 (1.06-3.79) | .032 | 11 (19) | 1.37 (0.64-2.92) | .412 | 6 (38) | 3.31 (1.06-10.31) | .039 | 10 (34) | 2.37 (0.97-5.81) | .059 |
| Suicidal intent/ideas | 55 (81) | 2.89 (1.50-5.59) | .002 | --- | --- | --- | 14 (88) | 4.75 (1.03-21.90) | .046 | 18 (62) | 0.70 (0.30-1.62) | .400 |
| **Primary diagnosis**  Any diagnosis of mental illness | 35 (51) | 1.80 (1.06-3.05) | .029 | 21 (37) | 0.92 (0.51-1.65) | .771 | 8 (50) | 1.61 (0.58-4.48) | .357 | 17 (59) | 2.25 (1.02-4.99) | .045 |
| Affective disorder (bipolar disorder and depression) | 15 (22) | 0.82 (0.39-1.72) | .602 | 11 (19) | 1.26 (0.51-3.15) | .614 | 3 (19) | 0.67 (0.16-2.90) | .598 | 3 (10) | 0.20 (0.05-0.73) | .015 |
| Anxiety/Obsessive compulsive/Post-traumatic stress disorder | 7 (10) | 1.42 (0.56-3.60) | .456 | 4 (7) | 1.33 (0.41-4.27) | .633 | <3 | --- | --- | 6 (21) | 3.71 (1.23-11.18) | .020 |
| **Recent events**  Relationship break-up | 19 (28) | 1.47 (0.82-2.63) | .200 | 20 (35) | 2.32 (1.27-4.25) | .006 | 4 (25) | 1.21 (0.38-3.86) | .746 | 3 (10) | 0.35 (0.10-1.20) | .094 |
| Relationship problems | 19 (28) | 1.11 (0.62-1.99) | .722 | 16 (28) | 1.14 (0.61-2.15) | .673 | 4 (25) | 0.95 (0.30-3.03) | .926 | 7 (24) | 0.86 (0.35-2.11) | .740 |
| Housing problems (including having recently changed accommodation) | 4 (6) | 0.32 (0.11-0.92) | .035 | 7 (12) | 0.90 (0.39-2.10) | .811 | <3 | --- | --- | 6 (21) | 1.65 (0.63-4.34) | .308 |
| Workplace problems | 12 (18) | 1.44 (0.71-2.94) | .313 | 8 (14) | 1.35 (0.59-3.11) | .477 | <3 | --- | --- | 3 (10) | 0.84 (0.23-3.01) | .789 |
| **Service contact (at any time)**  Mental health services (CAMHS and/or adult services) | 40 (59) | 1.71 (0.90-3.24) | .100 | 31 (54) | 2.22 (1.12-4.38) | .022 | 11 (69) | 3.28 (0.93-11.53) | .064 | 20 (69) | 2.37 (0.88-6.41) | .088 |
| Social care or local authority services | 12 (18) | 0.74 (0.37-1.48) | .396 | 15 (26) | 1.48 (0.75-2.89) | .255 | 3 (19) | 0.89 (0.24-3.33) | .862 | 14 (48) | 3.37 (1.51-7.53) | .003 |

**Supplementary Table 2 (continued): Antecedents of suicide by specific types of suicide-related online experience, UK (2014-2016)**

| **Data items** | **Searching for information on suicide method (n=68)** | | | **Posting suicidal ideas online (n=57)** | | | **Visiting websites**  **(n=16)** | | | **Online bullying**  **(n=29)** | | |
| --- | --- | --- | --- | --- | --- | --- | --- | --- | --- | --- | --- | --- |
|  | **N (%)** | **Adjusted OR (95% CI)** | ***p* value** | **N (%)** | **Adjusted OR (95% CI)** | ***p* value** | **N (%)** | **Adjusted OR (95% CI)** | ***p* value** | **N (%)** | **Adjusted OR (95% CI)** | ***p* value** |
| **Service contact (at any time)**  Youth Offending Team or local police force | 16 (24) | 0.66 (0.36-1.20) | .173 | 13 (23) | 0.68 (0.35-1.33) | .260 | 5 (31) | 1.06 (0.36-3.14) | .922 | 15 (52) | 2.54 (1.18-5.51) | .018 |
| Looked after child | 3 (4) | 0.44 (0.13-1.49) | .186 | 9 (16) | 2.71 (1.18-6.24) | .019 | <3 | --- | --- | 5 (17) | 2.09 (0.72-6.10) | .176 |
| No service contact | 21 (31) | 0.82 (0.44-1.51) | .522 | 17 (30) | 0.55 (0.28-1.06) | .073 | 4 (25) | 0.54 (0.16-1.90) | .341 | 3 (10) | 0.21 (0.06-0.74) | .015 |

^1^ Adjusted by age, gender and presence of a mental health diagnosis.

^2^ Difference between those with and without the specific type of suicide-related online experience (e.g. online bullying).

^3^ Adjusted by age, gender, presence of a mental health diagnosis and being in education (i.e. were a school pupil/student).
