# Supplementary material for: Online harms? Suicide-related online experience: a UK-wide case series study of young people who die by suicide
Source: Psychol Med. 2022 May 19;53(10):4434–45. doi: 10.1017/S0033291722001258 (PMC10388316; doi:10.1017/S0033291722001258)
Supplement: Supplementary file 1 [file S0033291722001258sup.zip › S0033291722001258sup004.docx]

**Supplementary Table 3: Number and proportion of young people reporting different combinations of multiple suicide-related online experience**

|  | **Searching internet for information on suicide method** | **Communicating suicidal ideas or intent online** | **Visited websites that may have encouraged suicide** | **Victim of online bullying** |
| --- | --- | --- | --- | --- |
| **Searching internet for information on suicide method** | ** |  |  |  |
| **Communicating suicidal ideas or intent online** | 17 (13%) | ** |  |  |
| **Visited websites that may have encouraged suicide** | 14 (11%) | 6 (5%) | ** |  |
| **Victim of online bullying** | <=3 | 9 (7%) | <=3 | ** |
